# Supercritical Impregnation of PETG with *Olea europaea* Leaf Extract: Influence of Operational Parameters on Expansion Degree, Antioxidant and Mechanical Properties

**DOI:** 10.3390/polym16111567

**Published:** 2024-06-01

**Authors:** Noelia D. Machado, José E. Mosquera, Cristina Cejudo-Bastante, María L. Goñi, Raquel E. Martini, Nicolás A. Gañán, Casimiro Mantell-Serrano, Lourdes Casas-Cardoso

**Affiliations:** 1 Chemical Engineering and Food Technology Department, Faculty of Science, Wine and Agrifood Research Institute (IVAGRO), University of Cadiz, Avda. República Saharaui, s/n, 11510 Puerto Real, Spain; cristina.cejudo@uca.es (C.C.-B.); casimiro.mantell@uca.es (C.M.-S.); lourdes.casas@uca.es (L.C.-C.); 2 Centre de Recherche de Royallieu, Laboratoire Transformations Intégrées de la Matière Renouvelable (TIMR), Ecole Supérieure de Chimie Organique et Minérale (ESCOM), Université de Technologie de Compiègne, Rue du Docteur Schweitzer CS 60319, 60203 Compiègne, France; joseruiz@mi.unc.edu.ar; 3 Instituto de Investigación y Desarrollo en Ingeniería de Procesos y Química Aplicada (IPQA–UNC–CONICET), Av. Vélez Sarsfield 1611, Córdoba X5016GCA, Argentina; laura.goni@unc.edu.ar (M.L.G.); raquel.martini@unc.edu.ar (R.E.M.); nicolas.ganan@unc.edu.ar (N.A.G.); 4 Instituto de Ciencia y Tecnología de los Alimentos, Facultad de Ciencias Exactas, Físicas y Naturales, Universidad Nacional de Córdoba (ICTA–FCEFyN–UNC), Av. Vélez Sarsfield 1611, Córdoba X5016GCA, Argentina

**Keywords:** polyethylene terephthalate glycol, *Olea europaea* leaf extract, mechanical properties, antioxidant loading, expansion degree

## Abstract

PETG (poly(ethylene glycol-co-cyclohexane-1,4-dimethanol terephthalate)) is an amorphous copolymer, biocompatible, recyclable, and versatile. Nowadays, it is being actively researched for biomedical applications. However, proposals of PETG as a platform for the loading of bioactive compounds from natural extract are scarce, as well as the effect of the supercritical impregnation on this polymer. In this work, the supercritical impregnation of PETG filaments with *Olea europaea* leaf extract was investigated, evaluating the effect of pressure (100–400 bar), temperature (35–55 °C), and depressurization rate (5–50 bar min^−1^) on the expansion degree, antioxidant activity, and mechanical properties of the resulting filaments. PETG expansion degree ranged from ~3 to 120%, with antioxidant loading ranging from 2.28 to 17.96 g per 100 g of polymer, corresponding to oxidation inhibition values of 7.65 and 66.55%, respectively. The temperature and the binary interaction between pressure and depressurization rate most affected these properties. The mechanical properties of PETG filaments depended greatly on process variables. Tensile strength values were similar or lower than the untreated filaments. Young’s modulus and elongation at break values decreased below ~1000 MPa and ~10%, respectively, after the scCO_2_ treatment and impregnation. The extent of this decrease depended on the supercritical operational parameters. Therefore, filaments with higher antioxidant activity and different expansion degrees and mechanical properties were obtained by adjusting the supercritical processing conditions.

## 1. Introduction

In recent years, polymers have been prominently featured in the biomedical and pharmaceutical fields. One important focus of current research in the biomedical area is the exploration of biomaterials with suitable mechanical properties to achieve their purpose [1]. A variety of polymers have been proposed for medical applications. Given its unique characteristic as biomaterial, PETG (poly(ethylene glycol-co-cyclohexane-1,4-dimethanol terephthalate)) is being actively researched in tissue engineering, dentistry, optometry, orthopedics, and vascular health, among others [2]. This polymer is biocompatible, non-cytotoxic, and compliant with the food exposure standards of the FDA [3]. Furthermore, it is inexpensive and recyclable. In the literature, some articles were found using PETG as a novel thermoplastic thermo-responsive shape memory polymer [4,5,6], for the fabrication of 3D medical implants such as for maxillofacial bone reconstruction [7], and as a potential material for bone scaffolds using a filament-based extrusion additive manufacturing system [8]. However, scarce reports were found proposing PETG as a medical or drug delivery device loaded with bioactive compounds. Recently, Arany et al. proposed an implantable drug delivery system based on diclofenac sodium and PETG filaments prepared by fused deposition modeling 3D printing [9]. The supercritical impregnation of this polymer was first proposed by Cejudo-Bastante and collaborators who employed antioxidant compounds derived from mango (*Mangifera indica* L.) leaf extract to functionalize this polymer intended for food and biomedical applications [10]. Antioxidant compounds are recognized for their therapeutic potential due to their crucial role in mitigating the damaging effect of reactive oxygen species (ROS) associated with numerous diseases. Incorporating antioxidant compounds into 3D-printed structures provides several benefits, including developing patient-specific implants, prosthetics, and drug delivery systems. This integration offers opportunities for localized delivery, controlled release, and customization of antioxidant therapies [11].

Supercritical fluid technology is sustainable and environmentally friendly and offers the possibility to obtain new products. Among other solvents, carbon dioxide (CO_2_) holds particular significance due to its abundance as a waste product, and its ease in achieving the supercritical state (critical temperature = 31.1 °C and pressure = 7.4 MPa). One significant advantage of this technology is its ability to be scaled up effectively with suitable apparatus. There is a range of industrial-scale high-pressure equipment and accessories readily available from various companies, particularly in the pharmaceutical sector [12]. Nevertheless, most of the research is currently conducted on a laboratory scale. Despite this, due to its similarities to supercritical fluid extraction, the scale-up of which has already been explored, the potential for scaling up this process could also be viable [13]. Furthermore, supercritical CO_2_ (scCO_2_) can be recycled, making it a highly efficient option [14]. The environmental impact of supercritical fluid technology has been evaluated for drying and extraction processes. Supercritical drying has proven to be a more environmentally friendly alternative than conventional methods with reduced process time, energy consumption, CO_2_ emissions, and auxiliary chemicals [14]. Similarly, supercritical extraction processes have also shown lower impacts on categories such as global warming, human toxicity, and freshwater ecotoxicity potentials, making it a more sustainable option for obtaining valuable compounds [15]. In this context, this technology is fully aligned with the concept of bioeconomy and the goals outlined in the 2030 agenda by the United Nations General Assembly [16]. The main advantage of employing scCO_2_ for the preparation of active polymeric materials relies on the wide spectrum of materials that could be obtained by varying the operating conditions. The scCO_2_ density and solvent power, gas-type diffusivity, and viscosity could be “tunable” by adjusting temperature and/or pressure changes. These changes affect the complex balance between scCO_2_, bioactive compound, and polymer that determines the final loading. Moreover, scCO_2_ has a great influence on the polymer structure. The type of polymer, together with the applied pressure and temperature, largely determines the amount of CO_2_ that can be dissolved in the polymer. The sorption of CO_2_ by the polymer could lead to several effects, such as plasticization, expansion of the polymer matrix, recrystallization, or foaming [12]. At this point, the pressure drop rate in the depressurization phase is crucial [17]. As a result, different materials could be obtained from the same polymer depending on the operating conditions. On one hand, unaltered materials may be the preferred choice if a subsequent process, such as 3D printing, is required or if the impregnation is carried out in the material’s final form. On the other hand, porous or foamed materials could be suitable for biomedical applications as active ingredient loading and release platforms, tissue engineering, and wound healing [18].

scCO_2_ has been employed for both impregnating polymers and extracting natural compounds. The application of natural extract to impart antioxidant activity to an innovative material has the advantage of delivering a higher polyphenolic profile and exploiting the synergic effect of these compounds in their biological activity. The scCO_2_-assisted impregnation has demonstrated superior outcomes compared to conventional impregnation methods like solvent casting. Cejudo-Bastante and coworkers have observed a more selective impregnation, increased antioxidant and antimicrobial activities against *E. coli*, and improved mechanical performance in nanocellulose films loaded with mango leaf extract when prepared using supercritical technology instead of solvent casting [19].

In 2019, the European Union (EU) reported that approximately 4.6 million hectares were dedicated to *Olea europaea* tree cultivation, based on the latest results from the five-year EU survey. The four EU member states that have the largest areas of olive trees, in descending order, are Spain (55%), Italy (23%), Greece (15%), and Portugal (7%). Together, these four countries account for over three-quarters of the total EU olive tree cultivation area [20]. Twice a year, the olive trees are pruned to increase olive production and facilitate fruit collection, generating 11.8 million tons of biomass per year in Europe, of which only 30% are used. The leaves correspond to one-third of the weight of this biomass. Currently, the olive grove biomass is most often burned, resulting in economic costs and environmental risks. Under these circumstances, the utilization of olive leaves as raw material for the obtaining of added-value compounds is a promising option to strengthen the profitability of olive plantations [21,22,23]. Moreover, this practice promotes sustainable agricultural practices that save precious natural resources and reduce waste by recycling and reusing products and components of agrifood waste into new valuable products [24].

Olive leaves have been widely used in popular medicine to treat diseases like fever and other inflammation-related conditions. In recent years, several studies have demonstrated that olive leaf extract has in vivo and in vitro antioxidant, antihypertensive, cardioprotective, anti-inflammatory, and antimicrobial properties, among other beneficial activities [25]. The interest in natural extract as an alternative to conventional medications has continued to evolve in modern medicine. Therefore, natural medicinal products have the potential to provide an efficient, cost-effective, and safe alternative to conventional medicines, allowing patients to receive greater and quicker access to treatment [26].

In this research, supercritical fluids were employed to obtain ethanolic extract rich in polyphenols with high antioxidant capacity from dried *Olea europaea* leaves [27]. Later, this extract was employed for the supercritical impregnation of PET-PP for food packaging films [27,28,29,30] and PLA and TPU filaments intended for biomedical applications [31].

In this context, this work focuses on the supercritical impregnation of *Olea europaea* leaf extract into PETG filaments, evaluating the influence of different operating conditions (pressure, temperature, and depressurization rate) on the expansion degree, antioxidant activity, and mechanical properties of the resulting filaments. The main objective of this work is to contribute to the knowledge about the structural changes that a polymer can undergo after a supercritical treatment in order to optimize the selection of conditions according to the proposed final application.

## 2. Materials and Methods

### 2.1. Materials

PETG filaments of 1.75 mm of nominal diameter (Tg: 85 °C) were purchased from Amazon (Seattle, WA, USA). The *Olea europaea* leaves were provided by an olive producer (San José de Lora de Estepa Olivarera SCA Coop, Seville, Andalusia, Spain). They were dried at room temperature and stored in the dark before use.

Carbon dioxide (CO_2_, 99.99% purity) was purchased from Abelló Linde S.A. (Barcelona, Spain). The solvents, ethanol (food grade, 96% *v*/*v*), and trichloromethane (99.8% purity) were obtained from Panreac AppliChem (Darmstadt, Germany).

The reactant 2,2-diphenyl-1-picrylhidrazil (DPPH) for the antioxidant assays was purchased from Sigma-Aldrich (Steinhelm, Germany).

### 2.2. Methods

#### 2.2.1. Enhanced Solvent Extraction of *Olea europaea* Leaves

Extractions were carried out under static mode following the same procedure reported by Cejudo-Bastante et al. with slight modifications [27]. The extraction equipment was provided by Thar Technologies (model SF1000, Pittsburgh, PA, USA) equipped with a 1 L high-pressure vessel, temperature controllers, supercritical CO_2_ and cosolvent high-pressure pumps, an automatic back-pressure regulator (BPR), and a cyclonic separator. A filter paper cartridge containing 254 g of dried and triturated olive leaves was introduced into a 1 L high-pressure vessel together with 500 mL of ethanol. Then, the CO_2_ was pumped at a flow rate of 10 g min^−1^ until a pressure of 120 bar was reached. The system was maintained in discontinuous mode at 120 bar and 80 °C for 24 h. The final extract was stored at room temperature and covered from light for further characterization before the impregnation procedure.

The yield of the extraction was determined using a gravimetric method [31] and the antioxidant activity of the extract was measured by the DPPH assay described in Section 2.2.6.

#### 2.2.2. Supercritical Solvent Impregnation

**Scheme 1:**
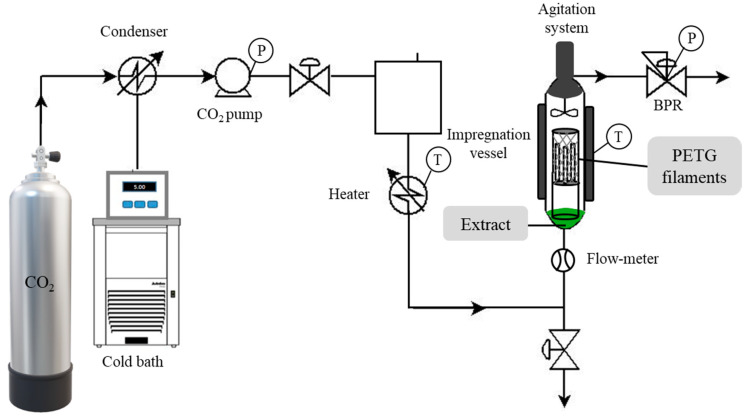
Schematic diagram of the supercritical solvent impregnation process (P = pressure, T = temperature, BPR = back pressure regulator).

The impregnation of olive extract into PETG filaments was performed using lab-scale high-pressure equipment provided by Thar Technologies (Pittsburgh, PA, USA, model SF500), as shown in Scheme 1.

The experiments were carried out in static mode following the previously reported procedure with minor modifications [31]. Different pressures (100, 250, and 400 bar), temperatures (35 and 55 °C), and depressurization rates (5 and 50 bar min^−1^) were evaluated. The impregnation was conducted using a 500 mL high-pressure vessel containing a magnetic paddle stirrer on top and a thermostatic jacket for controlling the temperature. Inside the vessel, 15 mL of olive ethanolic extract was added at the bottom to avoid direct contact with the polymer filaments. With this amount of extract, the experiments were carried out under supersaturation conditions with an excess of extract. Three PETG filaments approx. 100 mm long were vertically placed inside a metal cylinder covered with a metallic mesh. The system was heated at the set temperature and CO_2_ was pumped at 30 g min^−1^ up to the desired pressure. These conditions were maintained for 2 h, and the system was then depressurized at the corresponding rate. All impregnation runs were performed in duplicate.

#### 2.2.3. Expansion Degree (% *EXP*)

The expansion degree of the impregnated PETG filaments was calculated from Equation (1):

% *EXP* = (*d_f_* − *d_i_*)/*d_i_* × 100, (1)

where *d_i_* is the initial filament diameter and *d_f_* is the final filament diameter measured using a high-grade Vernier caliper after and before the supercritical process, respectively. These measurements were performed in duplicate. A design of experiment was performed to determine the influence of some process variables on the filament expansion degree (Section 2.2.8).

#### 2.2.4. Loading of *Olea europaea* Leaf Extract (*% L*)

The loading of the extract was measured using a UV-vis spectrophotometer (UV mini-1240, Shimadzu, Kyoto, Japan) at the maximum absorbance wavelength of 642 nm. A piece of impregnated filament (0.05 g) was dissolved into 3 mL of trichloromethane. The final concentration was calculated from the corresponding calibration curve in the same solvent (y = 341.24x − 0.0028, R^2^ = 0.9915, x = olive leaf extract concentration, g mL^−1^). Finally, the loading percentage (% *L*) was calculated from Equation (2):

% *L* = (*m_olive leaf extract_/m_pol_*) × 100, (2)

where *m_olive leaf extract_* is the mass of extract impregnated (g) and *m_pol_* is the mass of the polymer (g) employed in each assay. All experiments were performed in duplicate.

#### 2.2.5. Scanning Electron Microscopy

The morphological changes of impregnated PETG filaments were observed using a Nova NanoSEM 450 microscope (FEI, Hillsboro, OR, USA). The samples were cut into small pieces using a cutting plier and then were coated with a thin layer of gold (10 nm) using a Cressington Sputter Coater model 208 HR from Cressington Scientific Instrument (Watford, UK) for better conductivity and imaging.

#### 2.2.6. Antioxidant Activity

The DPPH free radical scavenging assay is a simple test where the DPPH radical is neutralized by donating electrons from the antioxidant compounds. The reaction is accompanied by changing the DPPH color at 515 nm from deep purple to pale yellow or discoloration [26]. This test was employed to determine the antioxidant activity of the impregnated PETG filaments following a previously reported procedure [31]. First, the antioxidant activity of the extract was determined as follows. A stock solution of DPPH 6 × 10^−5^ M was prepared using ethanol as solvent and stored in the dark before use. An aliquot of olive leaf extract (5, 10, 25, 50, 100, 250, and 500 μL) was added to the DPPH stock solution (v_f_ = 5 mL) and left to incubate in darkness at room temperature. After 2 h, the absorbance of the solution was measured at 515 nm, employing a UV-Vis spectrophotometer model Cary UV-Vis (Agilent Technologies, Santa Clara, CA, USA). The percentage of oxidation inhibition (% *OI*) of the olive leaf extract was calculated according to Equation (3):

% *OI* = (*A*_0_ − *A*_2*h*_)/*A*_0_ × 100, (3)

where *A*_0_ is the initial DPPH absorbance and *A*_2*h*_ is the absorbance of DPPH after 2 h of incubation with the extract. By plotting % *OI* vs. concentration of olive leaf extract (110–12,000 μg mL^−1^), a calibration curve was obtained (Equation (4)).

y = −2 × 10^−6^ x^2^ + 0.0351x − 2.1601, R^2^ = 0.993, (4)

The antioxidant activity of the impregnated PETG filaments was determined by placing a piece of filament (0.05 g) into 4 mL of DPPH stock solution. After 2 h, the absorbance at 515 nm was measured and the % *OI* of each sample was calculated according to Equation (3). The extract concentration with antioxidant activity finally impregnated in the PETG filaments was calculated from Equation (4). This concentration was expressed as g of impregnated antioxidant compounds per 100 g of polymer (% *AL*). In order to estimate the long-term stability, the antioxidant activity of impregnated filaments was evaluated after 12 months of storage in darkness at room temperature following the same procedure. All experiments were performed in duplicate.

A design of experiment was performed to determine the influence of some process variables on the filaments’ antioxidant activity (Section 2.2.8).

#### 2.2.7. Mechanical Properties

Tensile strength tests were conducted on the impregnated, scCO_2_-treated, and untreated filament samples in a universal testing machine Emic 23–5 S (Instron, Norwood, MA, USA) to assess the impact of both the high-pressure treatment and the incorporation of olive leaf extract on the mechanical properties of the resulting filaments. For all test cases, tensile tests were performed with samples of 150 mm, with an initial grip separation of 50 mm, and a crosshead speed of 700 mm min^−1^ until rupture. Filament thickness was precisely measured before all tests with a micrometer (0–25 mm ± 0.0001 mm, Asimeto, Germany). Tensile strength (*TS*), Young’s modulus (*E*), and elongation at break (*ε*) were meticulously recorded throughout the testing procedure and reported as the mean value ± standard deviation of at least 5 replicates.

A design of experiment was conducted to assess the impact of various process variables on the mechanical properties of the filaments (Section 2.2.8).

#### 2.2.8. Design of Experiments (DOE)

DOE is a statistical and mathematical tool used to conduct experiments and analyze data efficiently. In DOE, the levels of factors (experimental variables) are adjusted to ascertain the influence of individual factors and their interactions on the response [32]. A three-factor multilevel DOE was applied to assess the statistical effect of three operating variables on the impregnation process and the performance of impregnated PETG filaments. The selected response variables were the loading of *Olea europaea* leaf extract (% *L*), the antioxidant loading (% *AL*), expansion degree (% *EXP*), and the mechanical properties (tensile strength, Young’s modulus, and elongation at break). The selected process variables included pressure (A) at three different levels, as well as temperature (B) and depressurization rate (C) at two different levels each. In terms of the mechanical properties, the impact of depressurization rate was substituted with the impact of extract impregnation. The specific levels of each factor can be found in Table 1.

The selected temperature values range from 35 °C (close to the CO_2_ critical temperature of 31.1 °C) to 55 °C to prevent degradation of the bioactive compounds. Similarly, the chosen pressure values vary between 100 (close to the CO_2_ critical pressure of 74 bar) and 400 bar (close to the maximum limit of the equipment), with 250 bar included as an intermediate value. These values of pressure and temperature cover a wide range of CO_2_ density (337.2–972.2 kg m^−3^). The range of depressurization rates was determined according to the equipment capabilities, with options for both low and high rates.

The effect of each factor and all two-factor interactions on the response variables was statically determined by analysis of variance (ANOVA) using the software Statgraphics 18^®^ (StatPoint Technologies, Inc., Princeton, NJ, USA). Effects were considered significant for *p* < 0.05.

The single and multiple responses were optimized using the DOE desirability functions (*D*). The goal was to minimize % *EXP* response and to maximize % *AL*, *TS*, *E*, and *ε* responses with an impact factor = 3.

## 3. Results and Discussion

In the present work, the extraction of *Olea europaea* leaves was obtained using enhanced solvent extraction, as previously mentioned. The extraction yield was 10.7 ± 0.9% with a final dried weight concentration of 0.12 g mL^−1^. These results agreed with our earlier contributions [27,31]. This extract was employed for the supercritical impregnation.

The impregnated filaments were characterized by the expansion degree, extract loading, antioxidant activity, and mechanical properties. The discussion of the results is divided into four sections for better understanding.

### 3.1. Expansion Degree of Impregnated Filaments

The interaction between CO_2_ and the polymer matrix is favored by the good diffusivity of CO_2_, the presence of functional groups that are able to interact with CO_2_, and the amorphous nature of the polymer. Usually, this phenomenon could be observed as expansion or swelling since the CO_2_ sorption can increase the distance between polymer chains. In this context, conducting a preliminary analysis of a specific polymer’s CO_2_ sorption and swelling behavior typically yields valuable insights into how it will interact with the active ingredient during the diffusion process [12].

In particular, the interaction between PETG and CO_2_ is highly favored by the presence of carbonyl moieties in the ester functionalities and its amorphous nature (Tg = 85 °C) [2]. Previous studies have shown that the solubility of CO_2_ in PETG is quite high, 12.6 wt.% at 60 bar and 35 °C [33].

**Figure 1:**
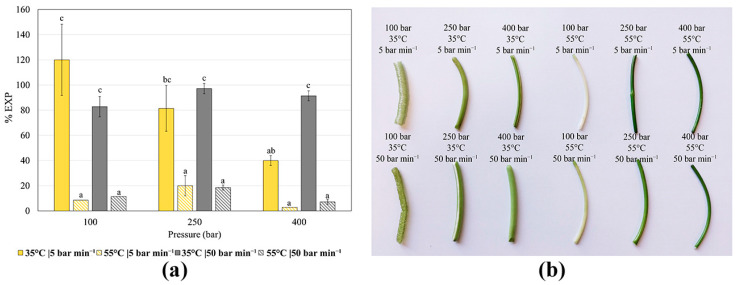
(**a**) Expansion degree (% *EXP*) of PETG filaments impregnated at 100, 250, and 400 bar, 35 and 55 °C, and depressurized at rates of 5 and 50 bar min^−1^. Different letters show significant differences among samples (Tukey test, α = 0.05). (**b**) Picture of the impregnated PETG filaments under the studied supercritical conditions.

The values of the expansion degree and images of the PETG filaments impregnated at each supercritical condition are shown in Figure 1.

The degree of expansion was significantly impacted by temperature, with higher values observed at 35 °C compared to 55 °C. Specifically, the values ranged from 40 to 120% at 35 °C and 3 to 20% at 55 °C (Table S1, Supplementary Material). The minimum degree of expansion (3%) was obtained at 400 bar and 55 °C (*D* = 0.9988), while the highest expansion (140%) was obtained at 100 bar and 35 °C using 5 bar min^−1^ of depressurization rate (Figure S1a, Supplementary Material). The influence of pressure and depressurization rate on polymer swelling was found to be more complex and will be further discussed.

Two phenomena can be observed from Figure 1b. Firstly, the green color of the filaments confirms successful extract impregnation, as the pristine filaments are transparent. Secondly, some filaments appeared foamed and deformed, especially those treated at 100 bar and 35 °C, while others were expanded without structural damage when treated at 250–400 bar and 35 °C. Additionally, some filaments showed less expansion when treated at 55 °C. In contrast with previous reports, the swelling effect of PETG filaments impregnated with mango leaf extract was lower at 35 °C than at 75 °C at 100 bar, reaching higher values (250% of expansion) [10]. These differences could be a consequence of the greater plasticizing effect of working at 75 °C, a temperature approaching the glass transition temperature of PETG.

**Figure 2:**
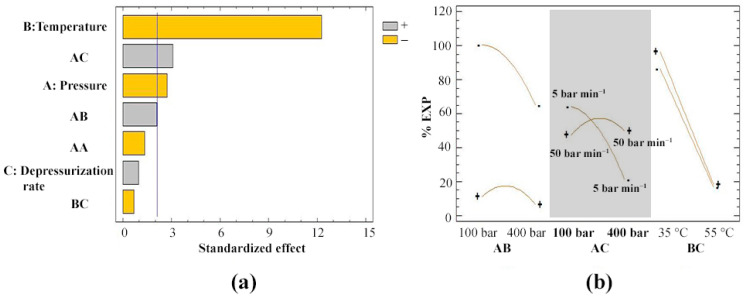
(**a**) Pareto standardized diagram for the expansion degree (% *EXP*) of PETG filaments. Process variables: A: pressure (bar), B: temperature (°C), C: depressurization rate (bar min^−1^). Note: the vertical line defines the significant effects that affect the process at a confidence level of 95%. (**b**) Effect of significant binary interaction (shaded) between process variables on % *EXP*. Note: The interactions between AB and BC were not statistically significant according to ANOVA testing (*p*-value > 0.05).

To better understand the effect of each process variable on the expansion of PETG filaments, a statistical analysis was performed. Figure 2 shows the obtained Pareto chart and the binary interactions between the studied variables.

As can be seen in Figure 2a, the polymer expansion was negatively affected by the temperature (*p*-value < 0.001) and the pressure (*p*-value = 0.0151), and positively affected by the combination of pressure–depressurization rate (*p*-value = 0.0076).

According to Figure 1b, the expansion of the polymer led to foamed filaments. The foaming process involves two basic steps: the sorption or dissolution of CO_2_ in the polymer matrix under pressure to form a polymer/gas solution, and the bubble nucleation and growth upon reduction of pressure [34]. The temperature and pressure impact the first step, the sorption of CO_2_ in the polymer matrix. The increase in pressure and temperature produced less expanded filaments (Figure S2a, Supplementary Material). At higher temperatures, the solubility of CO_2_ in the polymer decreases together with the bubble nucleation, producing a less foamed polymer than at lower temperatures. However, uncontrolled bubble nucleation sites are detrimental to foaming [34]. This phenomenon can explain the negative effect of pressure on polymer expansion degree. When the pressure increases, more fluid is dissolved in the polymer matrix, causing more plasticization and viscosity reduction. In this way, more nuclei are generated and more cells with smaller sizes are produced. Similar behaviors were observed for PLA when the pressure increased from 70 to 200 bar at 35 °C and for PS (polystyrene) from 180 to 380 bar at 80 °C [35].

The binary interaction between pressure and depressurization rate has a positive influence on polymer expansion. As shown in Figure 2b, slower depressurization rates at low pressure produced more foamed polymers, while the opposite occurred at high pressure. The change in pressure at 50 bar min^−1^ did not significantly affect the expansion degree (*p*-value = 0.9404). As mentioned before, the decompression stage is the driving force of the foaming process. During depressurization, the solubility of the polymer/gas mixture decreases, leading to the formation of nuclei and further expansion. In this step, nucleation is accompanied by and competes with the diffusion of CO_2_ in the plasticized polymer, resulting in pore growth. According to the single effect of pressure, more bubble nucleation is presented at high values, in comparison to lower pressures, which limits the bubble growth. Thus, if the venting is slow, the bubbles start to grow, but they collapse, leading to loss of expansion/densification. At lower pressures, where the nucleation sites are less, the slower venting produces more foamed polymer, since there is more time for pore growth and coalescence [36].

**Figure 3:**
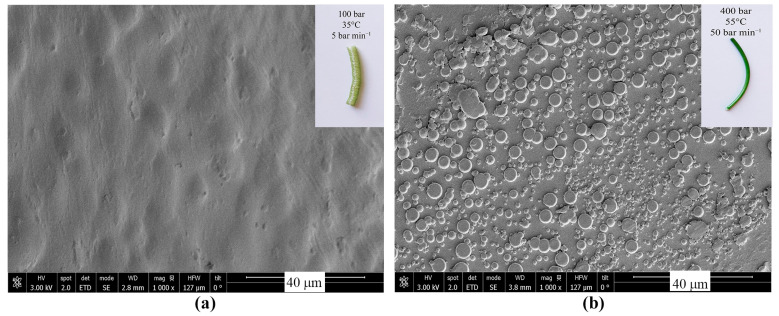
SEM images of the longitudinal section of PETG filaments impregnated at (**a**) 100 bar, 35 °C and depressurized at 5 bar min^−1^ and (**b**) 400 bar, 55 °C and depressurized at 50 bar min^−1^. (Magnification ×1000).

Figure 3 shows the morphology of PETG filaments impregnated under conditions that resulted in the highest and lowest levels of foaming, obtained by SEM.

Figure 3a shows the surface of PETG filaments impregnated at 100 bar, 35 °C and depressurized at 5 bar min^−1^. In these conditions, the filaments presented the highest foaming with an expansion degree of 120%. As can be seen, few and large bulges are observed, associated with few nuclei sites and high pore growth, as mentioned in Section 3.1. In contrast, Figure 3b shows the surface of the filaments impregnated at 400 bar, 55 °C and depressurized at 50 bar min^−1^. The picture shows more and smaller bubbles according to the greater number of nucleation sites and poor growth, resulting in a less foamed filament.

### 3.2. Impregnation of Olea europaea Leaf Extract into PETG Filaments

The analysis of the impregnation of a natural plant extract is quite complex due to the compound heterogeneity. According to a previous study, *Olea europaea* leaf extract is mainly composed of polyphenols belonging to the families of oleuropeosides (such as oleuropein and verbascoside), flavones (such as luteolin-7-glucoside and apigenin-7-glucoside), flavonols (such as rutin), and substituted simple phenols (such as hydroxytyrosol and caffeic acid) [27]. Moreover, carbohydrates and pigments such as chlorophylls are also presented in ethanolic extract [37,38].

**Figure 4:**
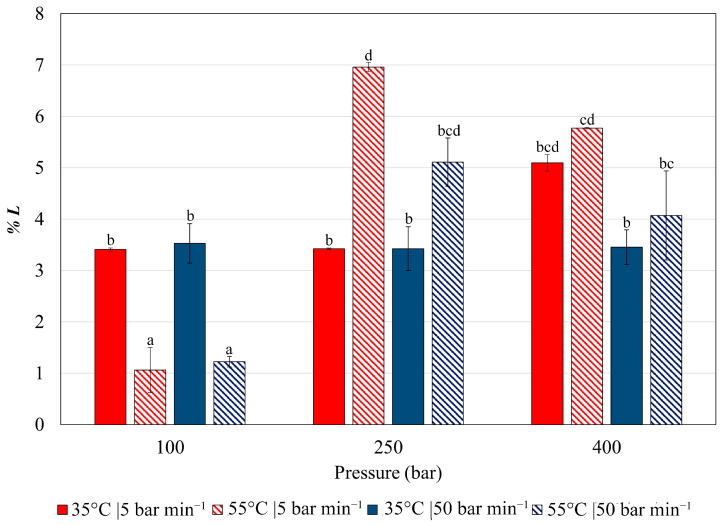
Loading of *Olea europaea* leaf extract (% *L*) of PETG filaments impregnated at 100, 250, and 400 bar, 35 and 55 °C, and depressurized at rates of 5 and 50 bar min^−1^. Different letters show significant differences among samples (Tukey test, α = 0.05).

Figure 4 shows the loading of *Olea europaea* leaf extract (% *L*) into PETG filaments, which were obtained by supercritical impregnation under different conditions of pressure, temperature, and depressurization rate.

The % *L* values ranged between 1.06 and 6.96% (Table S1, Supplementary Material). Generally, the highest values were obtained at 250 and 400 bar, while the lowest ones were achieved at 100 bar. These % *L* values are considerably higher than other results previously reported using the same polymer. Cejudo-Bastante et al. studied the supercritical impregnation of PETG filaments with mango leaf extract at 100–400 bar and 35–75 °C, obtaining loading values from ~0.1 to ~0.35% [10]. A high impregnation loading is promoted when the partition coefficient favors the polymer matrix against the supercritical fluid phase [39]. The mango leaf extract is also a complex mixture of different compounds, mainly represented by polyphenols such as mangiferin (C2-β-D-glucopyranosyl-1,3,6,7-tetrahydoxyxanthone) [40]. Due to the fact that this xanthone is a hydrophobic compound with an intermediate polarity [41,42], its partition during the impregnation process is favored to the scCO_2_ fluid phase (in comparison with the polymer matrix), resulting in low impregnation yields. In contrast, oleuropein, the major component of *Olea europaea* leaf extract, is highly hydrophilic [27,43,44]. Thus, its partition is preferential to the PETG matrix rather than to the scCO_2_ phase, yielding higher impregnation loadings.

**Figure 5:**
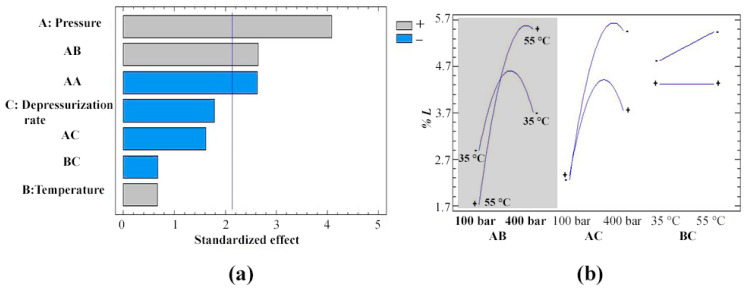
(**a**) Pareto standardized diagram for the loading of *Olea europaea* leaf extract (% *L*) into PETG filaments. Process variables: A: pressure (bar), B: temperature (°C), C: depressurization rate (bar min^−1^). Note: the vertical line defines the significant effects that affect the process at a confidence level of 95%. (**b**) Effect of significant binary interaction (shaded) between process variables on % *L*. Note: The interactions between AC and BC were not statistically significant according to ANOVA testing (*p*-value > 0.05).

According to the statistical analysis, the effects of the pressure and the pressure–temperature binary interaction on the scCO_2_ impregnation were significant (Figure 5a). The influence of pressure on % *L* is shown in Figure S2b, Supplementary Material.

The positive effect of pressure (factor A) on the % *L* values (*p*-value = 0.0010) can be explained by the increase in CO_2_ density with pressure, resulting in greater solvating power. This phenomenon increases the solubility of the solutes in the fluid phase, and consequently, the gradient of concentration between the fluid phase and the polymer, favoring the impregnation. Similarly, higher olive leaf extract loading values were found with the pressure increase (100–400 bar) in the supercritical impregnation of PLA and TPU filaments [31]. When PETG filaments were employed for the impregnation of mango leaf extract, higher extract loadings were obtained with the pressure increase (100 to 400 bar) [10].

The combined effect of pressure and temperature (factor AB, *p*-value = 0.0183) showed that high temperatures favored the impregnation loadings at high pressures but were disfavored at low pressures (Figure 5b). When the temperature increases at isobaric conditions, two contraposed phenomena occur. On one hand, the CO_2_ density decreases, disfavoring the impregnation, and on the other hand, the solute vapor pressure increases, promoting the loading. The predominant effect depends on the working pressure [45]. At low pressures (100 bar), the increase in temperature from 35 to 55 °C decreased ~3 times the % *L* values at any depressurization rate. In this condition, the dominant effect is the strong CO_2_ density drop (700.1 vs. 337.2 kg m^−3^), losing solvent power and reducing the solute concentration in the fluid phase. But at high pressure (400 bar), higher temperatures induced high % *L* values. Here, the CO_2_ density slightly decreased (972.2 vs. 906.85 kg m^−3^), the solute vapor pressure enhancement becoming the prevailing factor.

### 3.3. Antioxidant Activity

The bioactivity of the impregnated filaments was determined by measuring the antioxidant activity. The relevance of the determination of this activity relies on the fact that the oxidative processes promoted by free radicals take part in the physiology of very common diseases, like diabetes, high blood pressure, and atherosclerosis, among others [46].

In this work, the antioxidant activity was determined through a colorimetry reaction with an organic radical. The results of the oxidation inhibition *(*% *IO*) at the studied operation conditions are presented in Table S1, Supplementary Material. The % *OI* values ranged from 7.65 to 66.55%. The highest % *OI* values were obtained at 35 °C and all pressures, and 100 bar and 55 °C.

**Figure 6:**
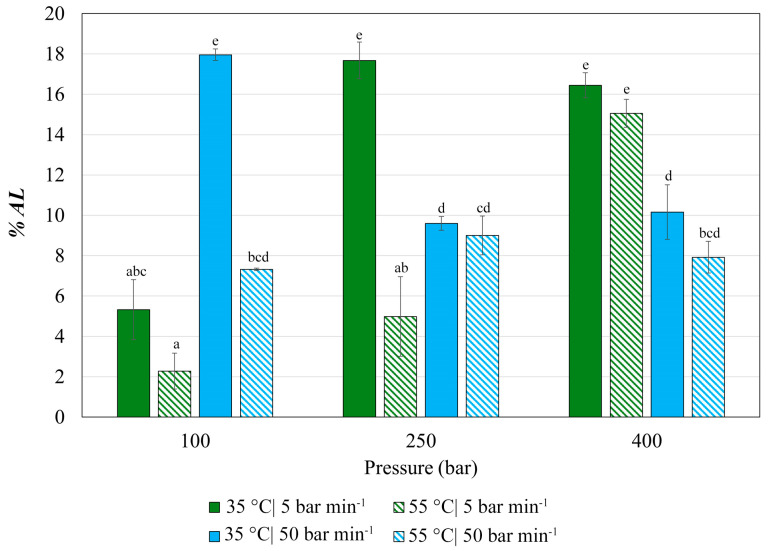
Antioxidant loading (% *AL*, g antioxidant per 100 g of polymer) into PETG filaments at 100, 250 and 400 bar, 35 and 55 °C, and depressurized at rates of 5 and 50 bar min^−1^. Different letters show significant differences among samples (Tukey test, α = 0.05).

The content of antioxidant compounds impregnated in the polymer filaments (% *AL*) was calculated from Equation (4) and is represented in Figure 6.

As shown in Figure 6, the content of antioxidant compounds impregnated in the polymer filaments ranged from 2.28 g (at 100 bar, 55 °C, depressurized 5 bar min^−1^) to 17.96 g per 100 g of polymer (100 bar, 35 °C, depressurized at 50 bar min^−1^) corresponding to % *OI* values of 7.65 to 66.55%, respectively (Table S1, Supplementary Material). In general, higher % *AL* values were observed at 35 °C for any pressure and depressurization rate (full-color bars). The increase in pressure at 35 °C favors the impregnation of antioxidant compounds when the depressurization rate is low (full green columns). On the other hand, the effect of pressure is the opposite when depressurization is faster (full blue light bars). These results are the consequence of the complex supercritical impregnation process. This process comprises three steps. The first includes the dissolution of the active compound in the supercritical fluid phase, then the diffusion of the resultant solution into the polymer matrix, and finally, the depressurization step [47]. In this process, the active compound–scCO_2_, active compound–polymer and polymer–scCO_2_ interactions compete and the prevalent one defines the final impregnation loading. Furthermore, the behavior of the polymer under supercritical conditions, like swelling and plasticizing, also affects the impregnation.

The optimal conditions to achieve the highest % *AL* were 400 bar and 35 °C, with the depressurization rate of 5 bar min^−1^ (*D* = 0.9721, Figure S1b, Supplementary Material). This operation condition did not correspond to those where the highest % *L* values were obtained. This result was also found previously when olive and mango leaf extracts were employed as sources of bioactive compounds in the impregnation of PLA, TPU, and PETG filaments [10,31,48]. The absence of a correlation between the total extract loading and the antioxidant loading could be related to two factors. The first could be the complex composition of these extracts with compounds of different sizes and polarities that determine their different solubilities and affinity with CO_2_ and the polymer matrix. In this way, there will be compounds (with and without antioxidant capacity) impregnated under the different impregnation conditions with more efficiency than others. The second one could be the underestimation in the quantification method for the determination of % *L* values and the overestimation in the % *AL,* since these values are calculated from the % *OI* values. It is known that the antioxidant activity of a natural extract could result from a synergistic effect among the antioxidants present [49].

The antioxidant loadings found in the current work are highly superior to those previously reported using 3D-printable filaments and natural extracts. Under similar conditions, the antioxidant loading for PLA filaments impregnated with olive leaf extract was 0.2–0.5 mg per g of polymer, while for TPU, the values were 2–3 mg per g of polymer at 100 and 400 bar and 55 °C, respectively [31]. Even considering the conditions where the highest loadings were obtained using PLA and TPU filaments, these values are 300 and 8 times lower than those reported in the current study under the same conditions for PLA (at 400 bar and 55 °C) and TPU (at 100 bar and 55 °C), respectively. When PET-PP films were employed, the antioxidant loading was considerably lower; 0.2 mg per g of polymer was determined at the best operating conditions (400 bar and 35 °C) [27]. The differences found in the antioxidant activity among these polymers could be the result of the affinity between all the antioxidant components soluble in the supercritical fluid phase and the polymers. PLA and PETG are polyesters with carbonyl and ether groups which could interact with the hydroxyl groups present in the polyphenols forming H-bonds. However, the aromatic rings of the main olive extract compounds could interact via π-stacking and Van der Waals forces with the terephthalate unit of PETG. Although PETG and TPU have similar potential sites of physical–chemical interactions, the porous structure found in PETG filaments may enhance accessibility and increase antioxidant loading. All in all, these interactions favor the impregnation of PETG against PLA and TPU. In the case of PET-PP films, the presence of a hydrophobic layer such as PP with hydrophobic methyl groups could hinder the affinity of polyphenols with this matrix, producing lower antioxidant loadings.

**Figure 7:**
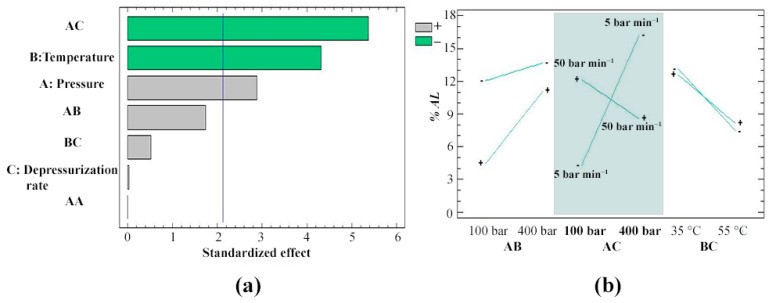
(**a**) Pareto standardized diagram for the antioxidant loading into PETG filaments (% *AL*, g antioxidant compounds per 100 g polymer). Process variables: A: pressure (bar), B: temperature (°C), C: depressurization rate (bar min^−1^). Note: the vertical line defines the significant effects that affect the process at a confidence level of 95%. (**b**) Effect of significant binary interaction (shaded) between process variables on antioxidant loading. Note: The interactions between AB and BC were not statistically significant according to ANOVA testing (*p*-value > 0.05).

In the present work, the statistical analysis showed that the antioxidant loading was strongly dependent on the impregnation process variables (Figure 7). Furthermore, new trends were observed in comparison with the % *L* values.

Based on Figure 7a, there is a negative correlation between the binary interaction of pressure and depressurization rate (factor AC), as well as temperature (factor B), with the % *AL*. However, pressure (factor A) showed a positive impact on the % *AL* (Figure S2c, Supplementary Material).

The positive effect of pressure (*p*-value = 0.0114) could be explained equally for % *L*. When pressure increases, the density of CO_2_ also increases, leading to improved solubility of antioxidant compounds in the fluid phase and facilitating their diffusion into the matrix. This ultimately results in higher levels of antioxidants being loaded into the polymer. A similar trend was observed by other authors, who obtained higher concentrations of polyphenols (extracted from *Olea europaea* leaves) impregnated in PET-PP films when the pressures increased from 100 to 400 bar at 35 °C [27,28,30]. On the other hand, the total phenolic content of the passion fruit bagasse compounds impregnated on corn starch aerogels increased when the pressure increased from 225 to 375 bar at 65 °C. The authors assumed that a rise in the partition coefficient, which is linked to the concentration of phenolic compounds in the scCO_2_, facilitated the impregnation into the polymer [50]. On the contrary, the opposite behavior was observed in the impregnation of methyl gallate (a polyphenol from mango extract) in PET-PP films when the pressure grew from 100 to 200 bar at 45 °C, indicating that there is a limit on the increase in solute solubility. Beyond that, the delicate affinity balance between the antioxidant compound and fluid phase or antioxidant compound and polymer is displaced to the supercritical phase, decreasing the impregnation efficiency [39].

Concerning the temperature (factor B), the significant negative influence (*p*-value = 0.0006) indicates that lower temperatures lead to higher antioxidant loadings (Figure S2c, Supplementary Material). This result was associated with both the high polymer expansion degree and CO_2_ density found at low temperatures. Similarly, the impregnation efficiency of total phenolic compounds from mango into polyester textile was negatively affected by the temperature when increased in the same range at 400 bar [49]. Rosales et al. also found that higher temperatures significantly decreased the antioxidant activity of PLA filament impregnated with mango leaf extract [48]. The authors attributed this result to the higher affinity of the antioxidant compounds with the supercritical solvent in comparison with the polymer, which could drag the solutes out of the vessel after the depressurization step.

A significant and negative interaction between pressure and depressurization rate (factor AC, *p*-value = 0.0001) was observed for the impregnation of antioxidants (Figure 7b). In this case, at 100 bar, slow depressurization rates resulted in low antioxidant loading, while at 400 bar, they led to higher values. The change in pressure at 50 bar min^−1^ did not significantly affect the antioxidant loading. (*p*-value = 0.2995).

The concentration of bioactive compounds in the fluid phase is lower at 100 bar compared to 400 bar due to the lower CO_2_ density. As mentioned in Section 3.1, bubble nucleation begins during the depressurization stage. At a slower depressurization rate, the antioxidant loading may be lower because the solutes along with the fluid phase are vented, while the pores in the polymer matrix are growing. Additionally, the poor concentration gradient at 100 bar may not favor impregnation into the polymer. However, the greater concentration gradient reached at 400 bar promotes bioactive loading. In these conditions, a slower decompression rate can encourage the interaction between the solutes and the polymer, improving the antioxidant loading. Furthermore, rapid pressure reduction rates can enhance the antioxidant loading, as the fast trapping or incorporation of solutes within the polymer prevents their removal into the supercritical fluid phase [12,45,51].

Few studies have been found in the literature investigating the impact of depressurization rate on the antioxidant activity of a natural extract-based material. Most of these studies focused on analyzing the effect of this variable on the impregnation loading of the natural extract as a whole. For instance, in the case of impregnating mango and olive extracts into polyester textile, PLGA-PEDOT scaffolds, and PET-PP films, it was found that similar depressurization rates (25, 50, and 100 bar min^−1^, respectively) under high pressures were also favorable [27,49,52]. The authors of these studies suggested that the rapid decrease in CO_2_ density during the depressurization step decreases the affinity with the solutes, resulting in the instant deposition of the compounds within the matrix.

In summary, the highest antioxidant loadings were achieved at 250 and 400 bar at a temperature of 35 °C with a depressurized rate of 5 bar min^−1^. The filaments depressurized at this rate were chosen for further analysis of their mechanical properties.

### 3.4. Mechanical Properties

The mechanical integrity of a new material is crucial, as it allows for the design of the necessary properties according to the intended final application. The mechanical properties of PETG filaments are summarized in Table 2. These properties were the tensile strength (*TS*), the Young’s or elastic modulus (*E*), and the elongation at break (ε).

The treatment with scCO_2_ as well as the extract impregnation significantly affected the mechanical properties of PETG filaments compared to the untreated filaments. The results of the multiple response optimization for impregnated filaments indicated that the impregnation conditions resulting in minimal changes were at 100 bar and 55 °C (*D* = 0.8207), whereas the maximal changes were noted at 100 bar and 35 °C (Figure S1c, Supplementary Material).

The tensile strength is the maximum tensile stress that a material carries, while the Young’s modulus or elastic modulus is a measure of the stiffness of the material [53,54]. According to Table 2, the *TS* values of filaments treated at supercritical conditions, whether impregnated or not, were similar or lower than those of the untreated filaments (*TS* value = 36.3 MPa). Similar results were found by other authors; for example, the *TS* values of LLDPE films after impregnation with carvone at 35 °C and a CO_2_ density of ~700 kg m^−3^ decreased ~15% compared to the neat films (38.4 MPa for neat films vs. 32.8 MPa for impregnated films) [55]. Other authors observed that the *TS* values of PLA films decreased ~78% by the supercritical fluid impregnation of thymol at 120 bar and 40 °C (from 58 MPa for neat films to 13 MPa for impregnated films) [56]. By contrast, when olive leaf extract was impregnated into PET-PP films, the *TS* values were higher than the control film (66.5 kN at 400 bar and 55 °C vs. 56.5 kN) [30]. The observed differences may be attributed to the structure of the impregnated PETG filament where the pores, as opposed to the film form, could facilitate the incorporation of extract. This, in turn, decreases interchain interactions, weakening the polymer.

On the other hand, the *E* values of filaments treated at supercritical conditions decreased in almost all cases below ~1000 MPa (>47% decrease) in comparison with the untreated filament (*E* value = 1890 MPa). Only the samples impregnated at 100 and 400 bar and 55 °C showed *E* values similar to the control filament (*p*-value 0.7684). When PLA films were employed for the impregnation of carvone at 98 bar and 60 °C, a significant decrease (98%) in the elastic modulus from 1592.3 MPa (untreated PLA) to only 36.3 MPa for carvone-loaded films was observed [57]. The same trend can be observed with the *E* values of LLDPE films, which were significantly affected by the impregnation under high-density conditions (345.4 MPa for neat film and 286.3 MPa for carvone-loaded films) [55].

The elongation at break is defined as the capability of a material to resist changes of shape without crack formation and gives information about mechanical resilience [56]. As shown in Table 2, the high elongation at break of neat PETG filament (*ε* = 43%) was considerably decreased (*ε* < 10%, an average decrease of 72%) after the scCO_2_ treatment and impregnation. This result indicates that the final material lost ductility. Only the *ε* values of pure scCO_2_-treated filaments obtained at 250 bar were similar to the control (*p*-value = 0.9784). A similar percentage decrease of ~65% was observed for LLDPE films (*ε* = 669%) after pure scCO_2_ treatment (*ε* = 219%) and impregnation with eugenol (*ε* = 249%) at 150 bar and 45 °C [58]. Nevertheless, the elongation at break of PETG filaments is comparable to those made from recycled PETG filaments that have been reinforced and filled with graphite for use in 3D printing [59].

**Figure 8:**
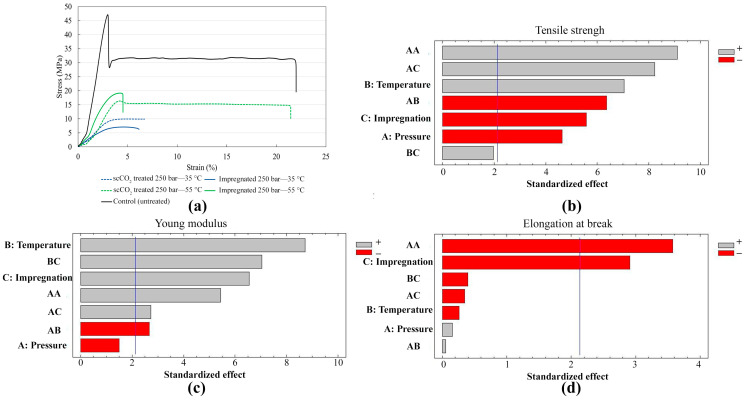
(**a**) Tensile stress–strain curves of untreated (neat), pure scCO_2_-treated, and olive leaf extract-impregnated PETG filaments (depressurization rate = 5 bar min^−1^). (**b**–**d**) Pareto standardized diagram for the mechanical properties of PETG filaments: (**b**) tensile strength, (**c**) Young’s modulus, and (**d**) elongation at break. Process variables: A: pressure (bar), B: temperature (°C), C: olive leaf extract impregnation. Note: the vertical line defines the significant effects that affect the process at a confidence level of 95%.

Figure 8a shows the tensile stress–strain curves of the untreated (neat) PETG filament and selected scCO_2_-treated and impregnated samples. The untreated PETG filament shows a ductile behavior with high yield and tensile strength, elastic modulus, and elongation at break. After the supercritical treatment, the ductile behavior in the elastic and plastic regions changes, being more evident at 55 °C than at 35 °C. A general decrease in the Young’s modulus and tensile and yield strength are observed. In addition, hardening is evidenced since the neck formation is no longer observed during elongation [60].

The scCO_2_ sorption and the presence of impregnated compounds can modify the original polymer arrangement, leading to decreased chain–chain interactions and an overall weakening of the material. It is worth noting that the extent of this decrease in the mechanical properties depends on the supercritical process variables. To understand how the supercritical process variables affected the mechanical properties of the filaments, a statistical analysis was performed. The Pareto diagrams for tensile strength, Young’s modulus, and elongation at break are shown in Figure 8b–d, respectively. The corresponding graphs depicting the effects of single variables and their binary interactions are presented in Figures S3 and S4 of the Supplementary Material, respectively.

*TS* values were significantly affected by the operation pressure and temperature, as well as by the incorporation of extract (impregnation). The tensile strength mean values decreased with increasing pressure from 100 to 250 bar, and conversely increased with pressure from 250 to 400 bar (Figure S3a). This may be explained in terms of the plasticizing effect of scCO_2_ and the extract and their effect on the polymer crystallinity. At a given temperature, scCO_2_ sorption increases with pressure (due to a higher fluid density), which may in turn weaken chain–chain interactions and increase chain mobility (plasticization). During depressurization, the original polymer structure may not be totally recovered, evidenced by a lower mechanical resistance. On the other hand, scCO_2_ sorption may also induce recrystallization, i.e., a rearrangement of polymer chains in more ordered structures, favored by the increased chain mobility, leading to a higher tensile strength. The complex behavior observed for the filament *TS*, with a minimum at an intermediate pressure, suggests that the first phenomenon (plasticization) prevails at lower pressure, while the latter (recrystallization) is dominant at higher pressure. Temperature showed a significant positive effect on *TS* values (*p*-value < 0.0001) (Figure S3a). Two main phenomena can be associated with temperature. On the one hand, at a given pressure, scCO_2_ sorption decreases with temperature (due to a density decrease); on the other hand, chain mobility is increased by temperature, leading to plasticization and thermal recrystallization. Results suggest that, in this case, the latter phenomena prevail. Finally, *TS* values for the impregnated samples are lower than for the pure scCO_2_-treated ones (*p*-value = 0.0001). In this case, the presence of the extract solutes induces a permanent plasticization, by hindering chain–chain interactions and reducing the recrystallization degree of the polymer.

Young’s modulus was significantly affected by the operation temperature and by the incorporation of extract (Figure S3b). *E* values increased with temperature, which can be related to the thermal recrystallization and subsequent strengthening effect mentioned above. Furthermore, impregnated filaments showed higher *E* values than pure scCO_2_-treated samples. In this case, the higher stiffness of impregnated samples may be due to a physical and low-range “crosslinking” effect induced by the presence of extract molecules interacting with the polymer chains by hydrogen bonding. These interactions may increase the polymer resistance to elastic deformation, without affecting the total tensile strength of the material, as discussed above.

Elongation at break was only affected by the impregnation of extract (Figure S3c), being higher for pure scCO_2_-treated samples. This result may be related to the above mentioned “crosslinking” effect due to solute–polymer interactions. However, as mentioned, almost all treated samples (impregnated or not) showed lower *ε* values than the untreated filament, suggesting that the supercritical treatment itself increases, to some degree, the polymer crystallinity.

The temperature (factor B) was one of the operating variables with the most significant effect on *TS* and *E* values (*p*-value < 0.0001). Higher values were found at 55 °C than at 35 °C. As discussed in Section 3.1, this variable had a significant impact on the degree of polymer expansion. It was observed that at 55 °C, the polymer exhibited lower expansion compared to 35 °C. Additionally, the temperature also influenced the loading of antioxidants in a contrary way; %*AL* was higher at 35 °C. In this sense, the *TS* and *E* values were higher when the polymer was less expanded and more loaded, indicating a predominant effect of the expansion degree. When the polymer is less expanded, greater interchain interactions are favored, contributing to strengthening the polymer.

All binary interactions between the studied variables also showed significant effects on the mechanical properties. The *TS* was greatly influenced by the combination of pressure and extract impregnation (factor AC, *p*-value < 0.001). Impregnated filaments processed at 100 bar showed lower *TS* values compared to samples subjected to pure scCO_2_ at the same pressure (Figure S4a, Supplementary Material). This weakening may be related to the higher expansion degree observed in impregnated samples. On the contrary, at 400 bar, the opposite effect is observed. At this pressure, the expansion degree is low, and the polymer loading is higher, which could help to strengthen the polymer via intermolecular interactions. A similar trend was found for the *E* values (factor AC, *p*-value = 0.0173) (Figure S4b, Supplementary Material).

The pressure–temperature binary interaction (factor AB) influenced the filament *TS* (*p*-value < 0.0001) and *E* (*p*-value = 0.0173). As previously discussed, these values were higher under low-density conditions (337.2 kg m^−3^ at 100 bar and 55 °C) (Figure S4a,b, Supplementary Material). However, these differences were not significant for *TS* at 400 bar, possibly due to the minimal changes in CO_2_ density at both temperatures (972.2 at 35 °C vs. 906.85 kg m^−3^ at 55 °C).

The *E* values were significantly affected by the combined effect of temperature–impregnation (factor BC, *p*-value < 0.001). The scCO_2_-treated filaments were more flexible, showing lower *E* values than the impregnated ones, especially at 55 °C (Figure S4b, Supplementary Material). This finding could be attributed to the higher plasticizing effect of scCO_2_. Furthermore, the inclusion of extract compounds in the polymer matrix could help to mitigate the structural weakening of the polymer, giving higher *E* values in comparison with the non-impregnated filaments.

In conclusion, there is evidence of a compromise between the high antioxidant levels and the mechanical properties of PETG filaments. The main factor influencing this compromise was the supercritical loading, while the expansion degree had a lesser impact. According to the results of the multiple response optimization, the filaments impregnated at 400 bar and 35 °C presented low expansion degree (40%), high antioxidant activity (*OI* values > 50%), and mechanical properties close to the control filament (*TS* value = 23.1 MPa, *E* value = 588.1 MPa, *ε* value = 4%) with a *D* = 0.7089 (Figure S1d, Supplementary Material). Therefore, this material could be suitable for further processing, such as 3D printing. On the other hand, the filament obtained at 250 bar and 35 °C with a similar antioxidant loading showed a high expansion degree (80%) and different mechanical performance (*TS* value = 7.4 MPa, *E* value = 283.4 MPa, *ε* value = 5%). This result may be counterbalanced using short carbon fiber-reinforced PETG filaments, which have demonstrated superior stress recovery [6]. However, although the mechanical properties are far from those of the control filament, they are still comparable to polymeric vascular stents like the PLA/PCL (poly(lactic acid)/poly(ε-caprolactone)) 70/30 mixture, with *TS* values ranging from 2 to 4.5 MPa, PC (polycarbonate) with *E* values of 200–240 MPa, and elongation at break like PLLA (poly(L-lactid acid)) and PLGA (poly(lactic-co-glycolic acid), with values of 2–6% [61]. Furthermore, the antioxidant activity of these impregnated filaments was assessed after 12 months post-preparation. Despite the mechanical properties, the findings revealed that 70% of the initial activity was retained, indicating a satisfactory level of stability. Hence, materials with varied antioxidant and mechanical properties could be produced by adjusting the supercritical processing conditions.

## 4. Conclusions

The supercritical impregnation of *Olea europaea* into PETG filaments was performed successfully. The filaments presented different expansion degrees, extract loading, antioxidant activity, and mechanical properties depending on the operating variables. Temperature was found to be the most influential variable, affecting the expansion degree and antioxidant loading the most, followed by pressure and the combination of both variables.

The impregnation of the extract had a strong impact on the mechanical performance of PETG filaments. However, tensile strength and elastic modulus were found to be significantly influenced by the supercritical operating variables. This study is the first to evaluate how supercritical conditions impact the mechanical properties of PETG filaments.

The findings suggest that PETG filaments with high antioxidant activity can be tailored for specific applications based on the chosen operating conditions. Nevertheless, further research is required to validate the in vitro and in vivo antioxidant effectiveness of these filaments, and more importantly, to ascertain their biocompatibility.

Possible perspectives in this area may involve evaluating the scaling up of the supercritical impregnation, analyzing its environmental impact, including variations in raw material sources or processing conditions, and assessing long-term sustainability and economic viability of the new material in comparison to conventional materials and processes. Overall, the results obtained in the present article as well as the perspectives set the stage for a comprehensive and multidisciplinary approach to understanding and optimizing the proposed process for the preparation of new antioxidant 3D-printable PETG filaments.

## Figures and Tables

**Table 1:** DOE for the loading of *Olea europaea* leaf extract (% *L*), the antioxidant loading (% *AL*), expansion degree (% *EXP*), and the mechanical properties: tensile strength (*TS*), Young’s modulus (*E*), and elongation at break (*ε*).

Factor	Variable	Levels	Responsive Variables
A	Pressure (bar)	100, 250, 400	% *L*, % *AL*, and % *EXP*
B	Temperature (°C)	35, 55	
C	Depressurization rate (bar min^−1^)	5, 50	
A	Pressure (bar)	100, 250, 400	*TS*, *E*, and *ε*
B	Temperature (°C)	35, 55	
C	Extract impregnation	−1 (no), +1 (yes)	

**Table 2:** Mechanical properties of PETG filaments treated at specified operation conditions, depressurization at a rate of 5 bar min^−1^ (±SD, *n* = 5). Tensile strength (*TS*), Young’s modulus (*E*), and elongation at break (*ε*).

Variables		scCO_2_-Treated			Impregnated with *Olea europaea* Extract		
Pres. (bar)	Temp. (°C)	*TS* (MPa)	*E* (MPa)	*ε* (%)	*TS* (MPa)	*E* (MPa)	*ε* (%)
100	35	34.6 ± 0.4	603.0 ± 43.7	6.0 ± 0.6	6.9 ± 0.5	202.0 ± 21.5	5.9 ± 1.3
	55	52.3 ± 2.3	1000.8 ± 67.3	3.8 ± 0.3	29.9 ± 1.3	1960.2 ± 248.8	4.0 ± 0.6
250	35	11.4 ± 0.7	172.0 ± 16.8	42.0 ± 18.6	7.4 ± 0.6	283.4 ± 50.2	5.0 ± 1.2
	55	16.7 ± 1.0	268.0 ± 27.6	38.5 ± 26.1	19.8 ± 0.5	953.2 ± 89.8	3.6 ± 0.4
400	35	22.9 ± 1.7	420.8 ± 67.2	6.2 ± 0.4	23.1 ± 19	588.1 ± 60.1	7.0 ± 1.5
	55	19.9 ± 1.8	329.2 ± 52.1	9.3 ± 3.5	22.4 ± 6.9	1875.2 ± 102.9	0.1 ± 0.4

Control PETG filament: *TS* = 36.3 ± 13.7 MPa, *E* = 1890.5 ± 388.8 MPa, *ε* = 43.1 ± 15.0%.

## Data Availability

The original contributions presented in the study are included in the article and Supplementary Material; further inquiries can be directed to the corresponding author.

## Supplementary Materials

The following supporting information can be downloaded at: https://www.mdpi.com/article/10.3390/polym16111567/s1, Table S1: Loading of *Olea europaea* leaf extract (% *L*, ±SD, *n* = 2), expansion degree (% *EXP*, ±SD, *n* = 2), oxidation inhibition (% *OI*, ±SD, *n* = 3), and antioxidant loading (% *AL*, ±SD, *n* = 3) of impregnated PETG filaments at the specified operation conditions.; Figure S1. DOE desirability functions of single responses: (a) % *EXP* (expansion degree), (b) % *AL* (g of extract impregnated per 100 g of polymer) and multiple responses (c) Mechanical properties (Tensile strength, Young modulus and elongation at break), (d) % *EXP*, % *AL* and mechanical properties (tensile strength, Young modulus and elongation at break). The goal was to minimize % *EXP* and maximize % *AL* and mechanical properties with an impact factor = 3.; Figure S2: Effect of significant single process variables (shaded) on: (a) Expansion degree, (b) % *L* (g of extract impregnated per 100 g of polymer) and (c) % *AL* (g of antioxidant compound impregnated per 100 g of polymer). Process variables: A: pressure, B: temperature, C: depressurization rate.; Figure S3: Effect of significant single process variables (shaded) on: (a) Tensile strength, (b) Young modulus and (c) elongation at break. Process variables: A: pressure, B: temperature, C: extract impregnation (−1 = non impregnated, +1 = impregnated).; Figure S4: Effect of significant binary interaction (shaded) between process variables on: (a) Tensile strength and (b) Young modulus. Process variables: A: pressure, B: temperature, C: extract impregnation.
