# Supplementary material for: Supercritical Impregnation of PETG with Olea europaea Leaf Extract: Influence of Operational Parameters on Expansion Degree, Antioxidant and Mechanical Properties
Source: Polymers (Basel). 2024 Jun 1;16(11):1567. doi: 10.3390/polym16111567 (PMC11174583; doi:10.3390/polym16111567)
Supplement: Supplementary file 1 [file polymers-16-01567-s001.zip › polymers-3018062-supplementary.pdf]

Article

Supercritical Impregnation of PETG with *Olea europaea* Leaf Extract: Influence of Operational Parameters on Expansion Degree, Antioxidant and Mechanical Properties

Noelia D. Machado [1,*], José E. Mosquera [2], Cristina Cejudo-Bastante [1], María L. Goñi [3,4], Raquel E. Martini [3,4], Nicolás A. Gañán [3,4], Casimiro Mantell-Serrano [1] and Lourdes Casas-Cardoso [1]

1 Chemical Engineering and Food Technology Department, Faculty of Science, Wine and Agrifood Research Institute (IVAGRO), University of Cadiz, Avda. República Saharaui, s/n, 11510 Puerto Real, Spain; cristina.cejudo@uca.es (C.C.-B.); casimiro.mantell@uca.es (C.M.-S.); lourdes.casas@uca.es (L.C.-C.)

2 Centre de Recherche de Royallieu, Laboratoire Transformations Intégrées de la Matière Renouvelable (TIMR), Ecole Supérieure de Chimie Organique et Minérale (ESCOM), Université de Technologie de Compiègne, Rue du Docteur Schweitzer CS 60319, 60203 Compiègne, France; joseruiz@mi.unc.edu.ar

3 Instituto de Investigación y Desarrollo en Ingeniería de Procesos y Química Aplicada (IPQA–UNC–CONICET), Av. Vélez Sarsfield 1611, X5016GCA Córdoba, Argentina; laura.goni@unc.edu.ar (M.L.G.); raquel.martini@unc.edu.ar (R.E.M.); nicolas.ganan@unc.edu.ar (N.A.G.)

4 Instituto de Ciencia y Tecnología de los Alimentos, Facultad de Ciencias Exactas, Físicas y Naturales, Universidad Nacional de Córdoba (ICTA–FCEFyN–UNC), Av. Vélez Sarsfield 1611, X5016GCA Córdoba, Argentina

* Correspondence: noelia.machado@uca.es

Table S1. Loading of *Olea europaea* leaf extract (% *L*, ± SD, n= 2), expansion degree (% *EXP*, ± SD, n= 2), oxidation inhibition (% *OI*, ±S D, n= 3), and antioxidant loading (% *AL*, ± SD, n= 3) of impregnated PETG filaments at the specified operation conditions.

Pressure (bar)	Temperature (°C)	Depressurization rate (bar min^{-1})	% *L**	% *EXP*	% *OI*	% *AL***
100	35	5	3.41±0.03	120.00±28.28	20.28±6.04	5.32±1.49
		50	3.53±0.38	20.00±8.08	66.55±1.01	17.96±0.28
	55	5	1.06±0.44	8.57±0.05	7.65±3.77	2.28±0.89
		50	1.22±0.10	11.43±0.10	28.29±0.25	7.32±0.06
250	35	5	3.42±0.01	81.43±18.18	65.48±3.02	17.68±0.91
		50	3.42±0.43	97.14±4.04	37.19±1.26	9.60±0.34
	55	5	6.96±0.09	20.00±8.08	18.86±8.05	4.98±1.97
		50	5.11±0.47	18.57±2.02	34.70±3.77	9.00±0.96
400	35	5	5.10±0.16	40.00±4.04	61.57±2.01	16.44±0.62
		50	3.45±0.34	91.43±4.04	39.15±5.03	10.16±1.36
	55	5	5.77±0.01	2.86±0.20	56.76±2.26	15.05±0.70
		50	4.07±0.86	7.14±2.02	30.60±3.02	7.92±0.79

* g of extract impregnated per 100 g of polymer
** g of antioxidant compound impregnated per 100 g of polymer

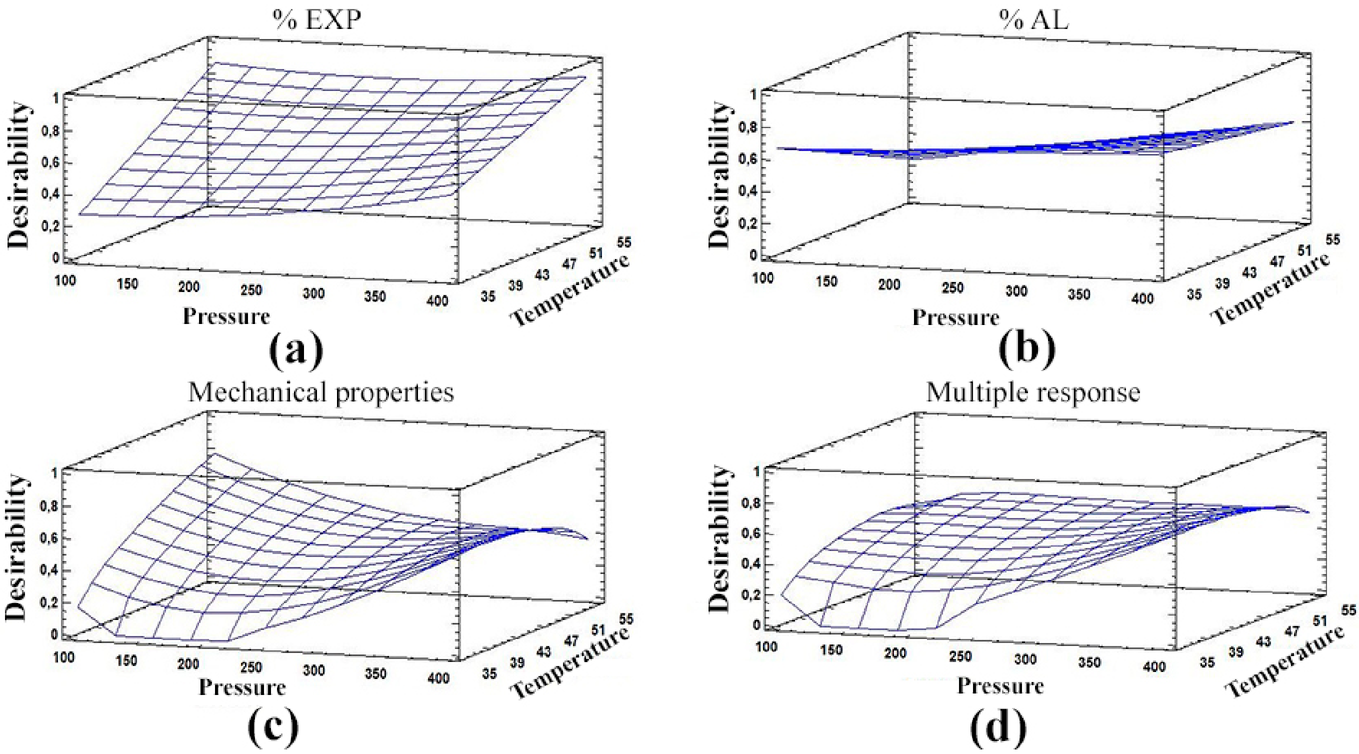

Figure S1. DOE desirability functions of single responses: (**a**) % *EXP* (expansion degree), (**b**) % *AL* (g of extract impregnated per 100 g of polymer) and multiple responses (**c**) Mechanical properties (Tensile strength, Young modulus and elongation at break), (**d**) % *EXP*, % *AL* and mechanical properties (tensile strength, Young modulus and elongation at break). The goal was to minimize % *EXP* and maximize % *AL* and mechanical properties with an impact factor= 3.

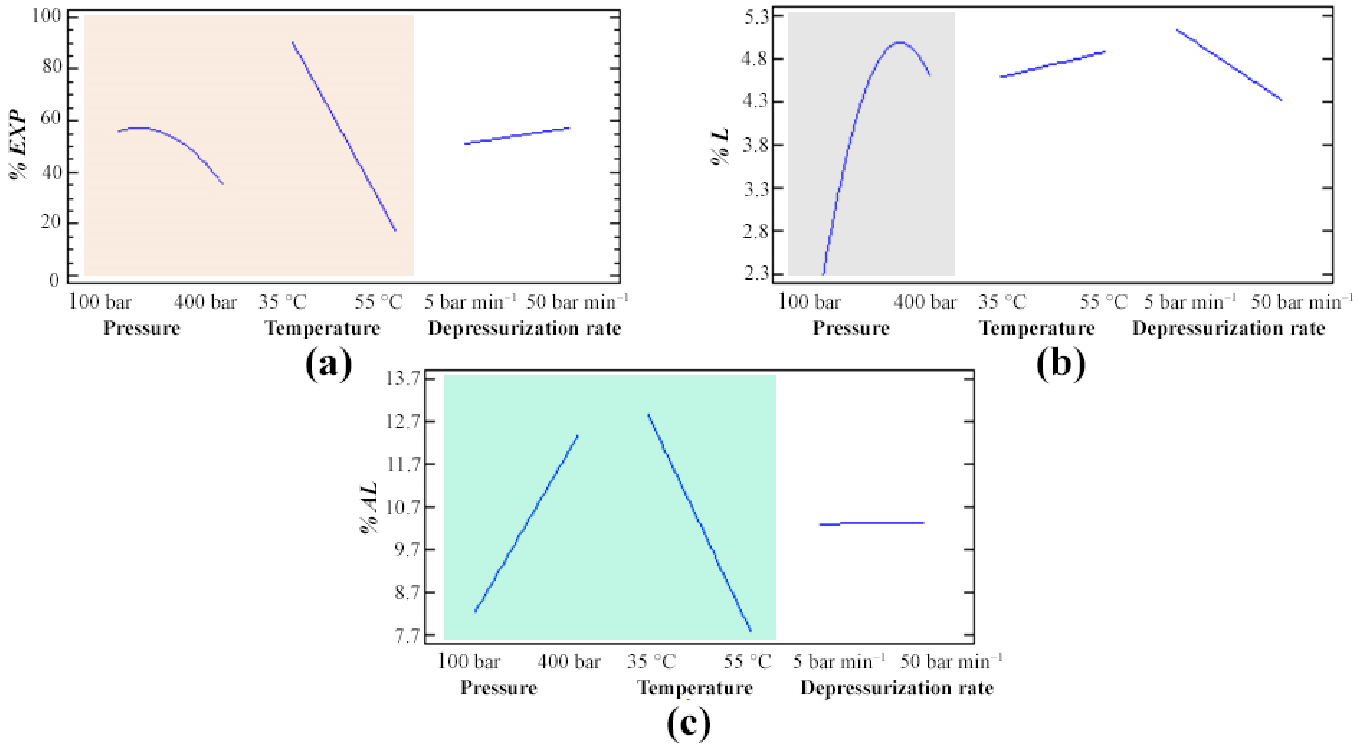

Figure S2. Effect of significant single process variables (shaded) on: (**a**) % *EXP* (expansion degree), (**b**) % *L* (g of extract impregnated per 100 g of polymer) and (**c**) % *AL* (g of antioxidant compound impregnated per 100 g of polymer). Process variables: A: pressure, B: temperature, C: depressurization rate.

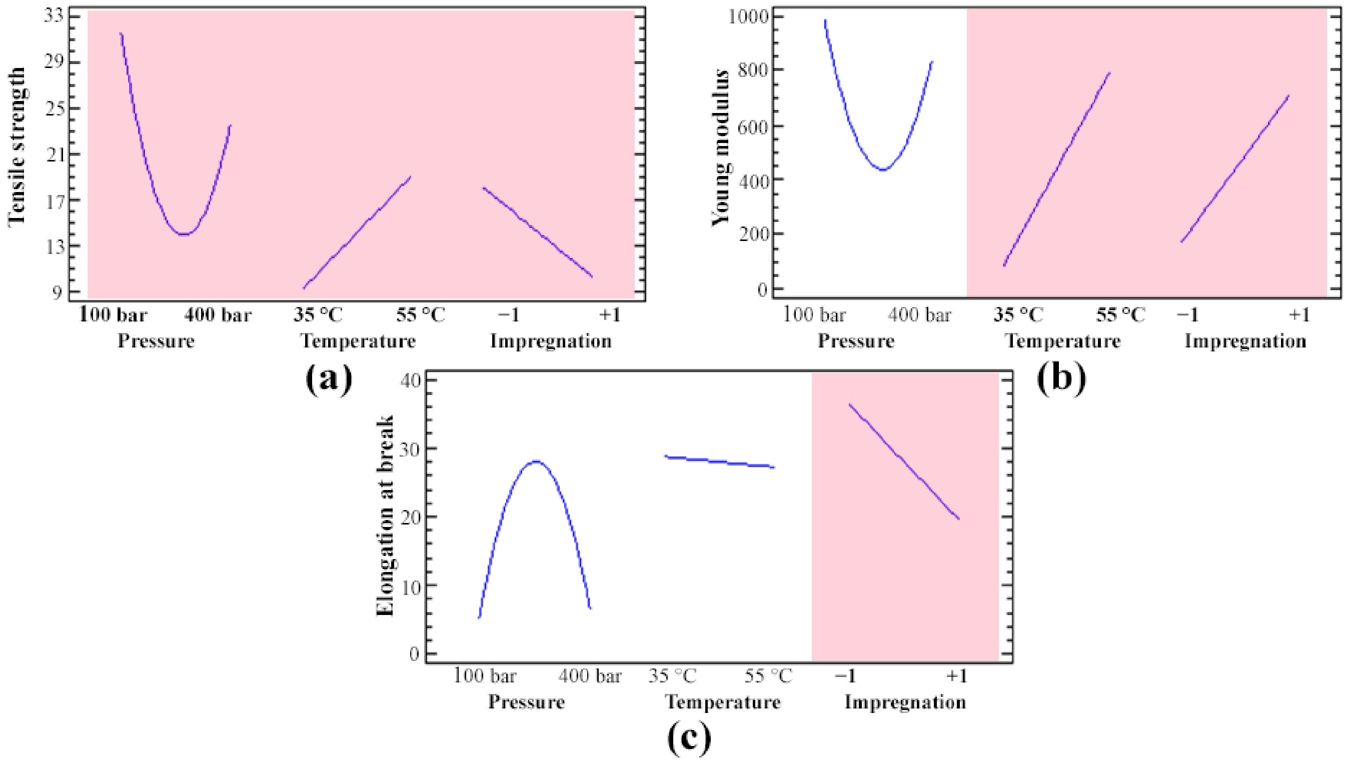

Figure S3. Effect of significant single process variables (shaded) on: (**a**) Tensile strength, (**b**) Young modulus and (**c**) elongation at break. Process variables: A: pressure, B: temperature, C: extract impregnation (−1= non impregnated, +1= impregnated).

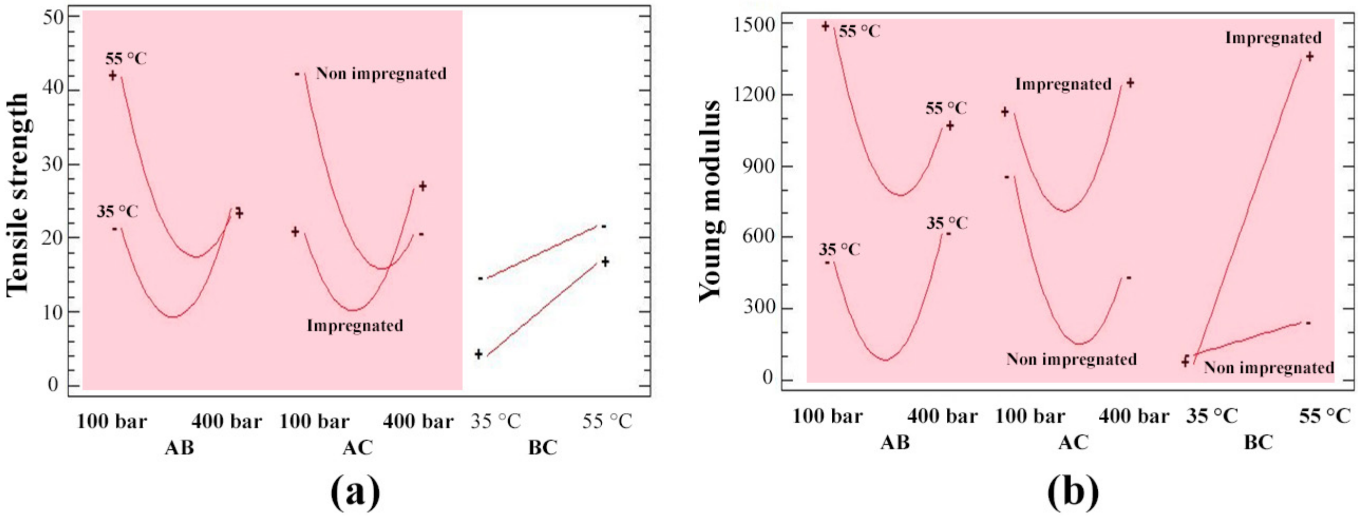

Figure S4. Effect of significant binary interaction (shaded) between process variables on: (**a**) Tensile strength and (**b**) Young modulus. Process variables: A: pressure, B: temperature, C: extract impregnation.
